# The association of high-intensity zones on MRI and low back pain: a systematic review

**DOI:** 10.1186/s13013-018-0168-9

**Published:** 2018-10-20

**Authors:** Masatoshi Teraguchi, Rita Yim, Jason Pui-Yin Cheung, Dino Samartzis

**Affiliations:** 1 Department of Orthopaedics and Traumatology, The University of Hong Kong, Hong Kong, Pokfulam SAR China; 2 Department of Orthopaedic Surgery, Wakayama Medical University, Wakayama, Japan; 3 Department of Orthopaedic Surgery, RUSH University Medical Center, 1611 W. Harrison Street, Suite 204G - Orthopaedic Surgery, Chicago, IL 60612 USA

**Keywords:** High-intensity zone, HIZ, spine, Lumbar, Pain, Systematic, Review, Outcomes, Phenotype, Disc, Degeneration, MRI

## Abstract

**Background:**

Magnetic resonance imaging (MRI) of the lumbar spine is commonly used to identify the source of low back pain (LBP); however, its use has been questionable. Throughout the years, numerous lumbar phenotypes (e.g., endplate abnormalities, Modic changes, black disc) have been studied as possible pain generators. High-intensity zones (HIZs) are of particular interest as they may represent annular tears. However, for over three decades, there has been heated debate as to whether these imaging biomarkers are synonymous with LBP. Therefore, the following study addressed a systematic review of the reported literature addressing the relationship of HIZs and LBP.

**Methods:**

A systematic review was conducted via MEDLINE, SCOPUS, Cochrane, PubMed, PubMed Central, EMBASE via Ovid, and Web of Science with the following search terms: “HIZ,” “high intensity zone,” or “high intensity zones” and “low back pain,” “pain,” “lumbago,” and/or “sciatica.” Specific exclusion criteria were also maintained. Two independent reviewers searched the literature, selected the studies, and extracted the data.

**Results:**

We identified six studies from our search strategy that met the inclusion criteria from a total of 756 possible studies. One cross-sectional population-based study and five comparison studies were identified, which provided information regarding the prevalence of HIZs. The prevalence of HIZs was 3 to 61% in subjects with LBP and 2 to 3% in subjects without LBP. Only three studies suggested a significant association between the presence of HIZ and LBP with or without sciatica.

**Conclusions:**

Our systematic review has found evidence that HIZs may be a possible risk factor for LBP; however, a mismatch of the clinical relevance of HIZs between studies still remains. The available evidence is limited by small sample size, heterogeneous study populations, and lack of standardized imaging methods for phenotyping. HIZs may be important lumbar biomarkers that demand further investigation and should be considered in the global imaging assessment of the spine, which may have immense clinical utility. Further large-scale studies with standardized imaging and classification techniques as well as the assessment of patterns of HIZs are necessary to better understand their role with LBP development.

## Background

Low back pain (LBP) is one of the most disabling conditions in the world and is associated with tremendous socioeconomic and health-care consequences [1–7]. Magnetic resonance imaging (MRI) of the lumbar spine is a frequently used imaging tool to characterize spine pathology and possibly identify the source of LBP [3]. Throughout the years, numerous lumbar phenotypes, such as endplate abnormalities, Modic changes, or black discs, have been identified and studied as potential pain generators to assist clinical decision-making and predicting outcomes [8–12]. However, there is often a discrepancy between what is noted on MRI and the clinical profile, leading to criticism regarding its utility in LBP management [13, 14]. One postulation for this mismatch is inappropriate phenotyping and its understanding. This may explain the unacceptable high incidence of failed spinal surgery and poor outcomes in LBP patients [8–10, 14–19].

**Fig. 1:**
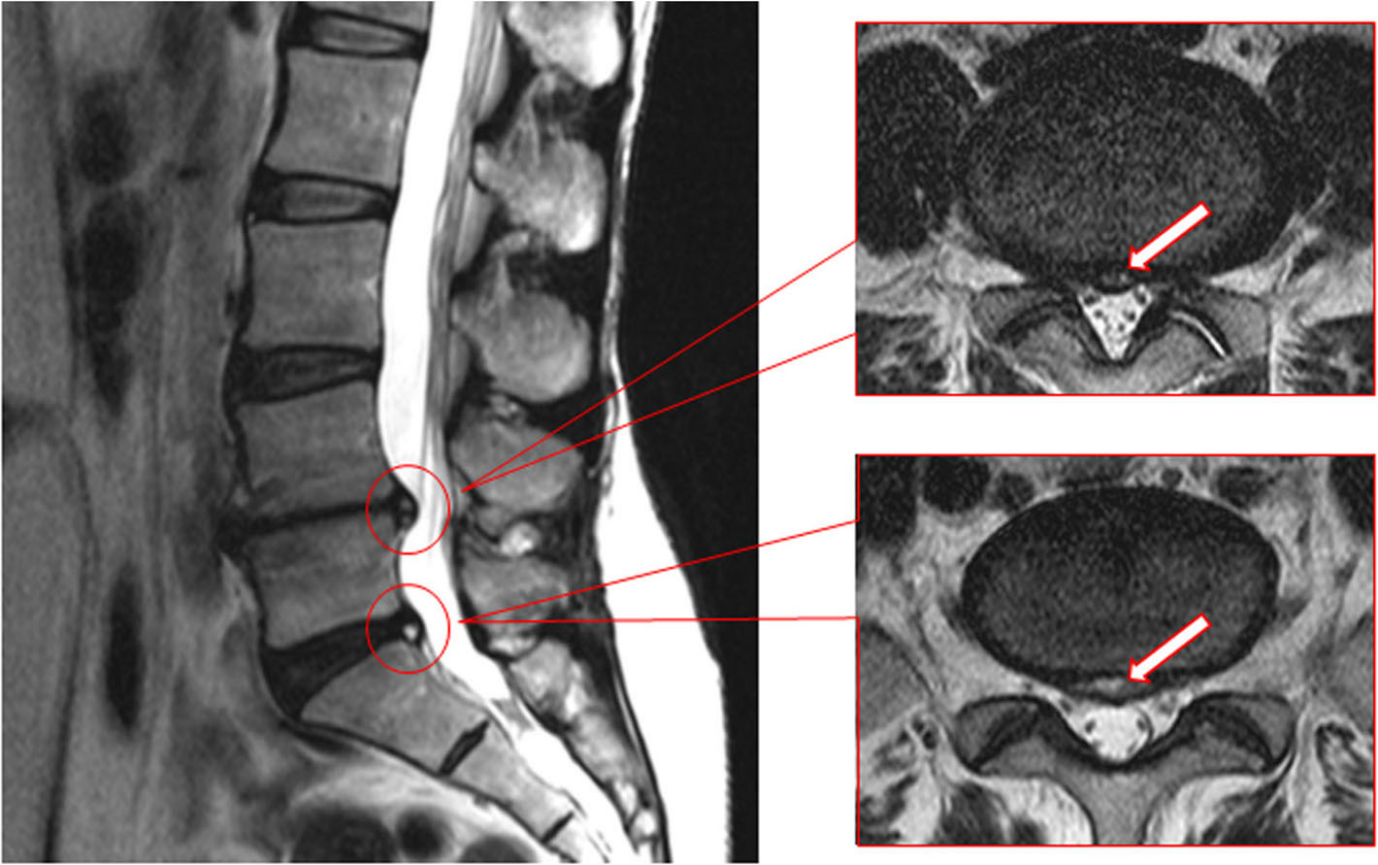
T2-weighted fast spin-echo sagittal and axial MR images demonstrating mid-posterior HIZs at the L4-5 and L5-S1 levels

**Fig. 2:**
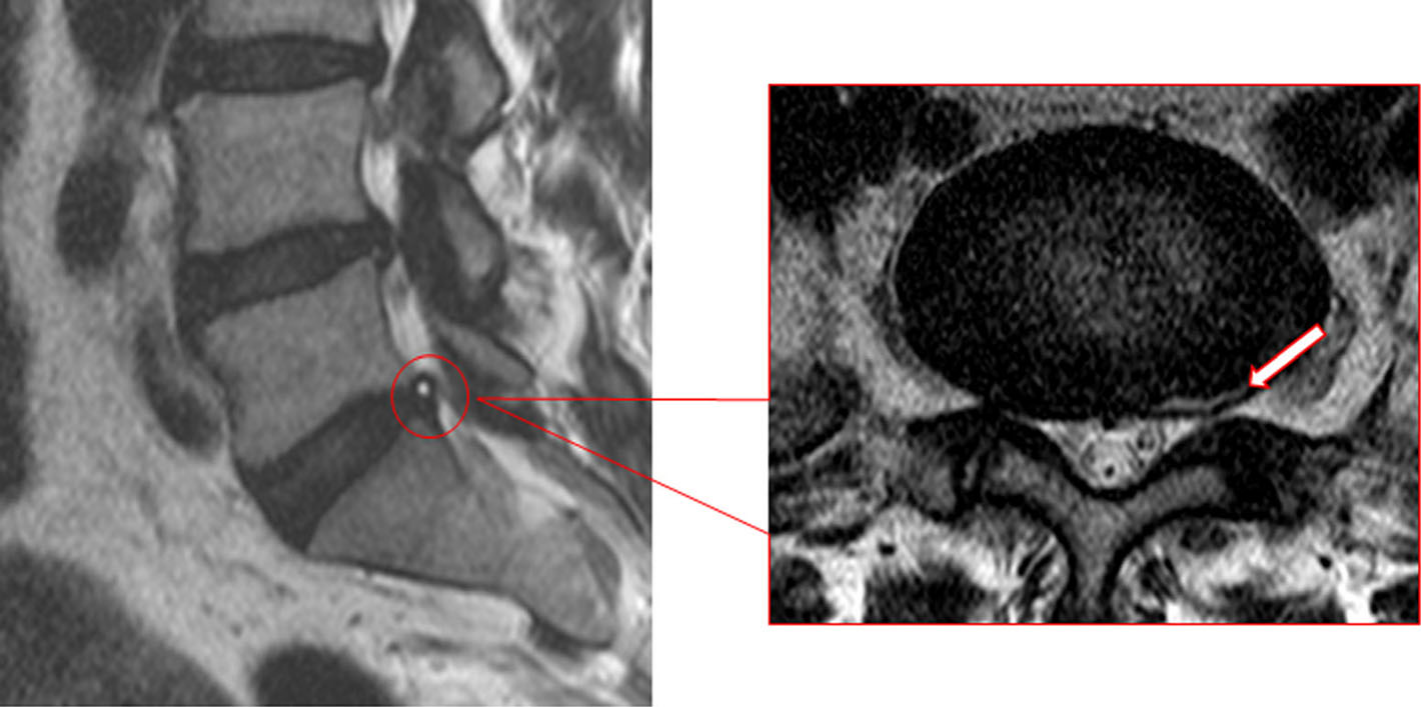
HIZ is noted in the posterolateral annulus of the L5-S1 disc

High-intensity zones (HIZs) are one such lumbar phenotype and are characterized as high-intensity regions of the annulus fibrosus of the intervertebral disc noted on T2-weighted MRI (Figs. 1 and 2). HIZs were initially reported in 1992 by Aprill and Bogduk [19] as potential imaging biomarkers related to a symptomatic disc. HIZs may be a specific marker of discogenic LBP because of its correlation with pain after provocation discography [19]. Due to the invasive nature of discography, non-invasive MRI became popularized for the identification of HIZs, which expressed annular tears. However, throughout the years, contradictory studies have surfaced to debate the clinical implications of HIZs [20–31]. This may be attributed to studies having no comparative symptomatic or control groups and utilizing heterogeneous populations.

In lieu of the above discrepancy of HIZs and their association with pain as well as the potential limitation of previous studies, the following study addressed a systematic review of the literature to address the association of HIZs with LBP.

## Methods

### Study search

A systematic review of the literature was performed in accordance with the Preferred Reporting Items for systematic review [32]. Two reviewers (MT, RY) independently searched the literature to identify potential articles based on pre-established exclusion and inclusion criteria. The literature search was conducted via MEDLINE, SCOPUS, Cochrane, PubMed, PubMed Central, EMBASE via Ovid, and Web of Science with the following search algorithm: “HIZ” “HIZs,” “high intensity zone” or “high intensity zones” and “low back pain” or “pain” or “lumbago” or “sciatica.” The databases were searched from their start of archiving until September 1 of 2017. The two reviewers also selected the articles and extracted the data.

### Inclusion and exclusion criteria

Only prospective cohort studies and comparative, cross-sectional studies were included in this systematic review due to their high level of evidence. Studies involving the following procedures and methodologies were excluded: retrospective study designs, case series/reports, review articles, non-English studies, only available abstract of studies, review article/ operational protocol, history for spinal surgery or invasive discography, cadaveric study, prevalence studies with insufficient information, and only symptomatic subject studies.

### Study selection

**Fig. 3:**
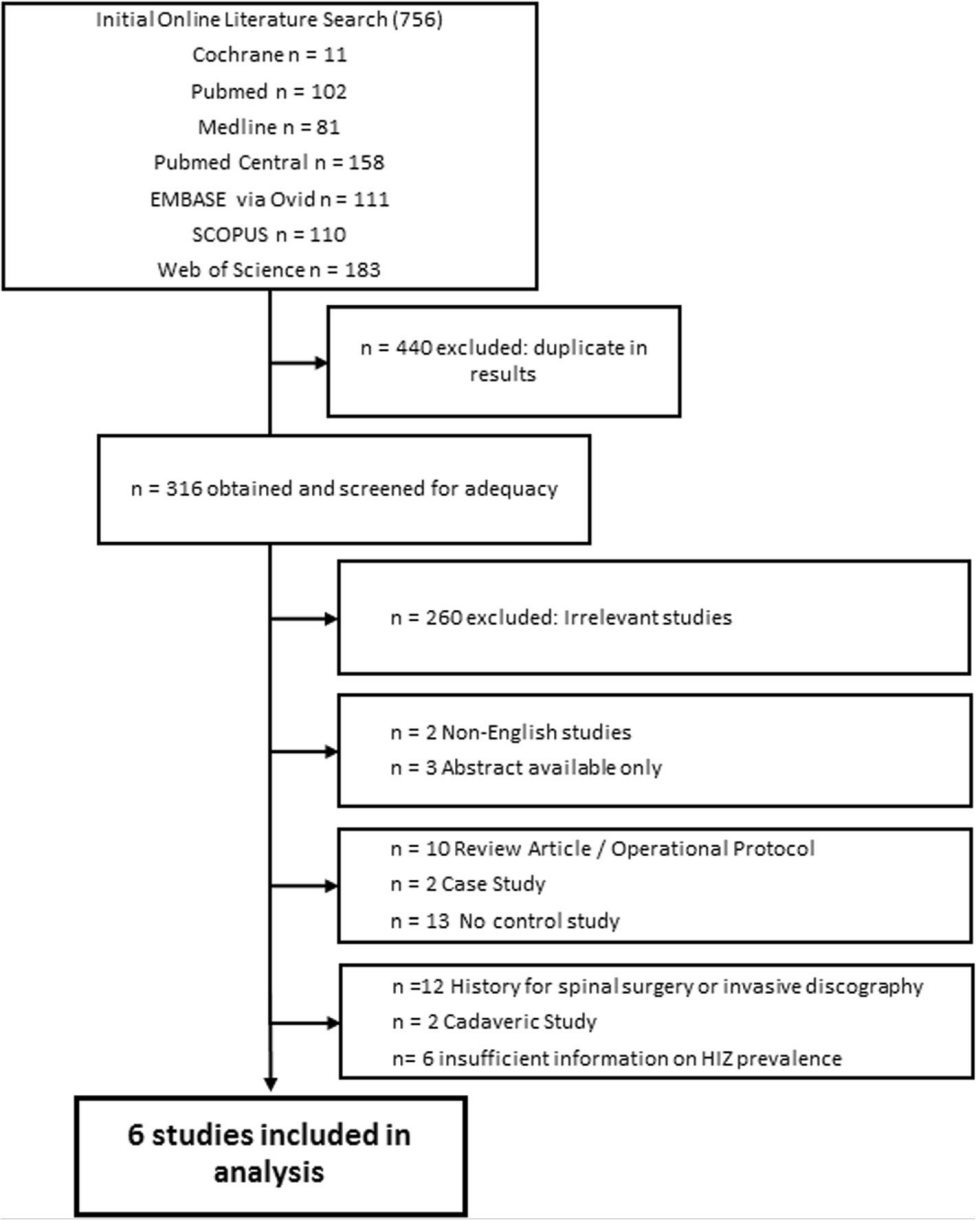
Flow diagram of literature search and included studies

The initial search retrieved 756 retrieved articles. After removing 440 duplicates, the titles and abstracts of 316 publications were screened. We excluded 260 citations due to irrelevant studies, 2 citations due to non-English studies, 3 citations due to only abstract availability, 10 citations due to review articles, 2 citations due to case reports, 13 citations due to no control study, 12 citations due to history for spinal surgery or invasive discography, 2 citations due to cadaveric study, and 6 citations due to insufficient information on HIZ prevalence (Fig. 3). Finally, full-text assessment resulted in 6 eligible articles included in the final analysis. The definition and diagnostic criteria for HIZs varied among the studies (Table 1).

**Table 1:** Summary of definition of high-intensity zones (HIZs) in the lumbar spine

Author	Year	Definition of high-intensity zones (HIZs)
Carragee et al.	2000	Central intensity of high-intensity signal was within 10% of the CSF intensity.
Hancock et al.	2012	High-intensity signal located in the substance of the posterior annulus fibrosus, which is brighter than the nucleus pulposus in T2-weighted images.
Takatalo et al.	2012	High-intensity signal located in the substance of the posterior annulus fibrosus, which is brighter than the nucleus pulposus in T2-weighted images
Wang et al.	2012	High-intensity signal located in the substance of the posterior annulus fibrosus, which is brighter than the nucleus pulposus in T2-weighted images. Three-dimensional localization method was described as follows: (a) posterior annular HIZ signs on sagittal T2-weighted MR images were distinguish; (b) target HIZ in the left, middle, or right part of the annulus fibrosus were located according to the relative position to mid-sagittal plane on scout view; and (c) a straight line was drawn across the midpoint of ventral edge and the midpoint of dorsal edge of the lumbar disc on sagittal view. The location of HIZs was confirmed in accordance with the relative position to the line and characterized as HIZs in superior, middle, or inferior annulus.
Liu et al.	2014	High-intensity signal located in the substance of the posterior annulus fibrosus, which is brighter than the nucleus pulposus in T2-weighted images. The posterolateral lesions were also included. If HIZ was evident in more than one sagittal image, the largest lesion was selected.
Yang et al.	2015	High-intensity signal located in the substance of the posterior annulus fibrosus, which is brighter than the nucleus pulposus in T2-weighted images.

*CSF* cerebrospinal fluid, *ROI* region of interest

### Statistical analyses

A meta-analysis was not performed due to the heterogeneity of the study design that would preclude pooling of studies. Qualitative assessment of the study parameters was performed. Lead author, study year, study design, characteristics, and outcome variables were extracted. HIZ prevalence rates of overall subjects with or without symptoms as well as per disc level, if available, were noted.

## Results

### Study characteristics

Of the six studies evaluated studies in this review, the year of publication ranged from 2000 to 2015. All six studies were prospective cohort or comparative studies. Table 1 shows the summary of definition of HIZs in the lumbar spine. Table 2 shows the study design, methodologies, and outcome of HIZs, such as prevalence of HIZs in symptomatic patients and controls, the highest prevalence at disc level, and association with LBP. All studies included both symptomatic patients and asymptomatic controls, with the reported mean age between 21 years to 50 years.

**Table 2:** Summary of study designs, methodologies, and outcome of high-intensity zones (HIZs) in the lumbar spine

Author	Year	Sample size (*N*)	Mean age (years)	Radiologic methods (MRI)	Prevalence	The highest prevalence at disc level	Association with LBP
Carragee et al.	2000	96	38			N/A	No
		Symptomatic, 42 Asymptomatic, 54	Symptomatic, 36 Asymptomatic, 40	1.5T anterior and posterior T2-weighted	Symptomatic, 25 (59%) Asymptomatic, 85 (24%)		
Hancock et al.	2012	60	37			N/A	No
		Symptomatic, 30 Control, 30		1.5T sagittal T1 and T2-weighted axial T2-weighted	Symptomatic, 18 (30%) Control, 7 (22%)		
Takatalo et al.	2012	554				N/A	No
		Symptomatic, 387 Asymptomatic, 167	21	1.5T sagittal and axial T2-weighted	Symptomatic, 13 (3.2%) Asymptomatic, 4 (2%)		
Wang et al.	2012	623				L4/5	Yes
		Symptomatic, 317 Asymptomatic, 306	50	1.5T sagittal T1-weighted sagittal and axial T2-weighted	Symptomatic, 115 (36%) Asymptomatic, 85 (28%)		
Liu et al.	2014	151	43			L4/5	Yes
		Symptomatic, 72 Asymptomatic, 79	Symptomatic, 44 Asymptomatic, 43	1.5T sagittal T1 and T2-weighted	Symptomatic, 33 (45.8%) Asymptomatic, 16 (20%)		
Yang et al.	2015	57	29			N/A	Yes
		Symptomatic, 25 Asymptomatic, 32	Symptomatic, 30 Asymptomatic, 28	3.0T sagittal and axial T1- and T2-weighted	Symptomatic, 17 (61%) Asymptomatic, 11 (32%)		

### Prevalence and clinical implications of HIZ

Three comparative studies indicated the significant association between HIZs and LBP [12–17] (Table 3). Wang et al. [14] reported a clinical series, which included 623 patients (337 males and 286 females, age 50.1 ± 15.4 years), and 200 patients (32.1%) exhibited HIZs in at least one disc. Furthermore,33 LBP patients had multi-segmental HIZs (5.3%) with 24 LBP patients also having HIZs in adjacent discs (3.9%) and reported that 57.5% of HIZs patients were symptomatic, which was significantly higher than the percentage of patients who exhibited no HIZs (*p* = 0.023) by Pearson chi-square tests. Although the incidence of LBP was higher when the HIZ disc level was lower (i.e., L4/5 or L5/S1) and when multiple HIZs were identified, the association was statistically insignificant. Yang et al. [15] studied HIZs in 57 patients (28 male and 29 female) with disc protrusions undergoing lumbar discectomy. This study suggested that 61% (17/25 patients) of those with HIZs also had LBP as compared to only 32% (11/32 patients) of patients without HIZs. The median prevalence rate of HIZs is 39.1% in symptomatic patients and 21.3% in asymptomatic controls. Liu et al. [15] similarly studied LBP symptomatic patients (*n* = 72) and asymptomatic controls (*n* = 79). The prevalence of HIZs in patients with LBP and controls without LBP was 45.8% and 20.2%, respectively. After being adjusted with cerebrospinal fluid intensity, the mean signal of HIZs in patients with LBP was significantly brighter than in asymptomatic patients (57.6 ± 14.0% vs. 45.6 ± 7.2%, *p* < 0.001) in the quantitative measurements by Student *t* test.

**Table 3:** Summary of the association between high-intensity zones (HIZs) and low back pain (LBP)

Author	Year	Association of subjects with HIZ and LBP
Carragee et al.	2000	Of the 42 symptomatic patients, 25 had HIZ: 1 patient had three HIZ discs; 6 patients had two HIZ discs; and 18 patients had one HIZ disc. The asymptomatic group had 13 HIZ discs in 13 of the 54 patients (24%). No reliably associated with HIZ of LBP.
Hancock et al.	2012	No significant differences in rates of MRI findings between controls with no and 1–2 past episodes of LBP.
Takatalo et al.	2012	HIZ occurred in similar frequencies in all clusters of LBP and back-related functional limitations. However, there is no significant association between HIZ lesions and LBP.
Wang et al.	2012	The LBP rate of HIZ patients was significantly higher than that of patients who exhibited no HIZ (57.5 vs. 47.8%, *p* < 0.05). There was no evidence for a correlation between LBP and spatial distribution of HIZ in disc (*p* > 0.05).
Liu et al.	2014	The mean signal of HIZ in symptomatic subjects was significantly brighter than in asymptomatic subjects (57.5 ± 14.0% vs. 45.6 ± 7.22%, *p* < 0.05). There was no statistical difference of area of disc and HIZ between the two groups. MRI index was found to be higher in symptomatic subjects comparing with asymptomatic subjects (3.94 ± 1.71 vs. 3.06 ± 1.50, *p* < 0.05).
Yang et al.	2015	LBP incidences were compared between the groups of HIZ (+) and HIZ (−). The data demonstrated that 60.7% (17/28 patients) of patients in HIZ (+) group were with LBP, while 20.7% (6/29 patients) of patients in HIZ (−) group were with LBP (*p* < 0.05)

On the other hand, three controversial results can be seen from larger scale population-based studies. One cross-sectional population-based study identified provided information regarding the prevalence of HIZs [21]. Based on this Northern Finland birth cohort of 554 subjects at 21 years of age, the prevalence of HIZs was reported as 3.2%. Takatalo et al. [21] found no association between HIZs and LBP in 554 Finnish young subjects. Similarly, Hancock et al. [24] reported that HIZs occurred in 30% of the patients with at least moderate pain of less than 6-week durations, and HIZs also occurred in 22% of the controls with no or one to two prior LBP episodes. Carragee et al. [20] suggested that the prevalence of HIZs in symptomatic subjects was 59% while the prevalence was 24% in the asymptomatic subjects. In addition, 33 (30.2%) of 109 discs were found to have a HIZ in the symptomatic group, as compared to only 13 (9.1%) of 143 discs in asymptomatic subjects. Further, this study noted that a high percentage of asymptomatic controls with degenerative disc changes were found to have HIZ, and these patients most often experienced pain with disc injection. The prevalence of HIZ was less than that of symptomatic participants, but the pain response on discography was equal in symptomatic and asymptomatic individuals.

### Disc level involvement

Only two studies thoroughly assessed disc level involvement. Wang et al. [22] reported the highest prevalence of HIZs to be at L4/5 or L5/S1. Similarly, Liu et al. [23] also suggested that HIZs were more frequently seen in L4/5, followed by L5/S1.

## Discussion

MRI is most commonly used in the diagnosis of patients with LBP [33]. However, there is often discrepancy and critique between the clinical profile and MRI findings [27, 33, 34]. HIZs are one of the MRI findings which may be a LBP generator. However, the association between HIZs and LBP has been under debate for the past two decades [19–31]. To our knowledge, there is no systematic review of the literature which addressed the association of LBP with HIZs in both symptomatic patients and controls. The purpose of this systematic review was to assess the association between patients with HIZs and without HIZs in relation to pain. Six prospective cohort or comparative studies were included in our systematic review [20–25], whereby we analyzed the prevalence of HIZs and clinical significance of HIZs with regard to LBP. Results from this review suggest that HIZs was more prevalent in subjects with LBP than in subjects without LBP in three comparative studies. However, the other studies could not find the significant association of LBP with HIZs.

Then, the main ongoing debate regarding HIZs is whether they are symptomatic or not. Currently, there is no consensus as reports have contradictory evidence and are based on limited evidence from studies without control groups, underpowered, or heterogeneous population studies [11–23]. Regarding some previous studies using discographic injection for HIZ, the relationship between the presence of lumbar HIZs with a concordant pain response on provocative discography was noted as a significant MRI biomarker of the diagnosis of discogenic LBP [11, 12]. On the other hand, Ricketson et al. [27] and Buirski et al. [34] were not able to demonstrate a statistically significant correlation between the presence of HIZs and pain concordant with the usual symptoms elicited during provocative discography [27, 34]. Furthermore, Carragee et al. [20] demonstrated that discographic injections provoked significant pain in approximately 70% of cases irrespective of whether patients had pain or not. This indicates that even though a discographic injection in a disc with an HIZ may produce significant pain, the disc may not be the cause of LBP [20]. Thus, provocative discographic studies are of limited use for determining symptomatology.

**Fig. 4:**
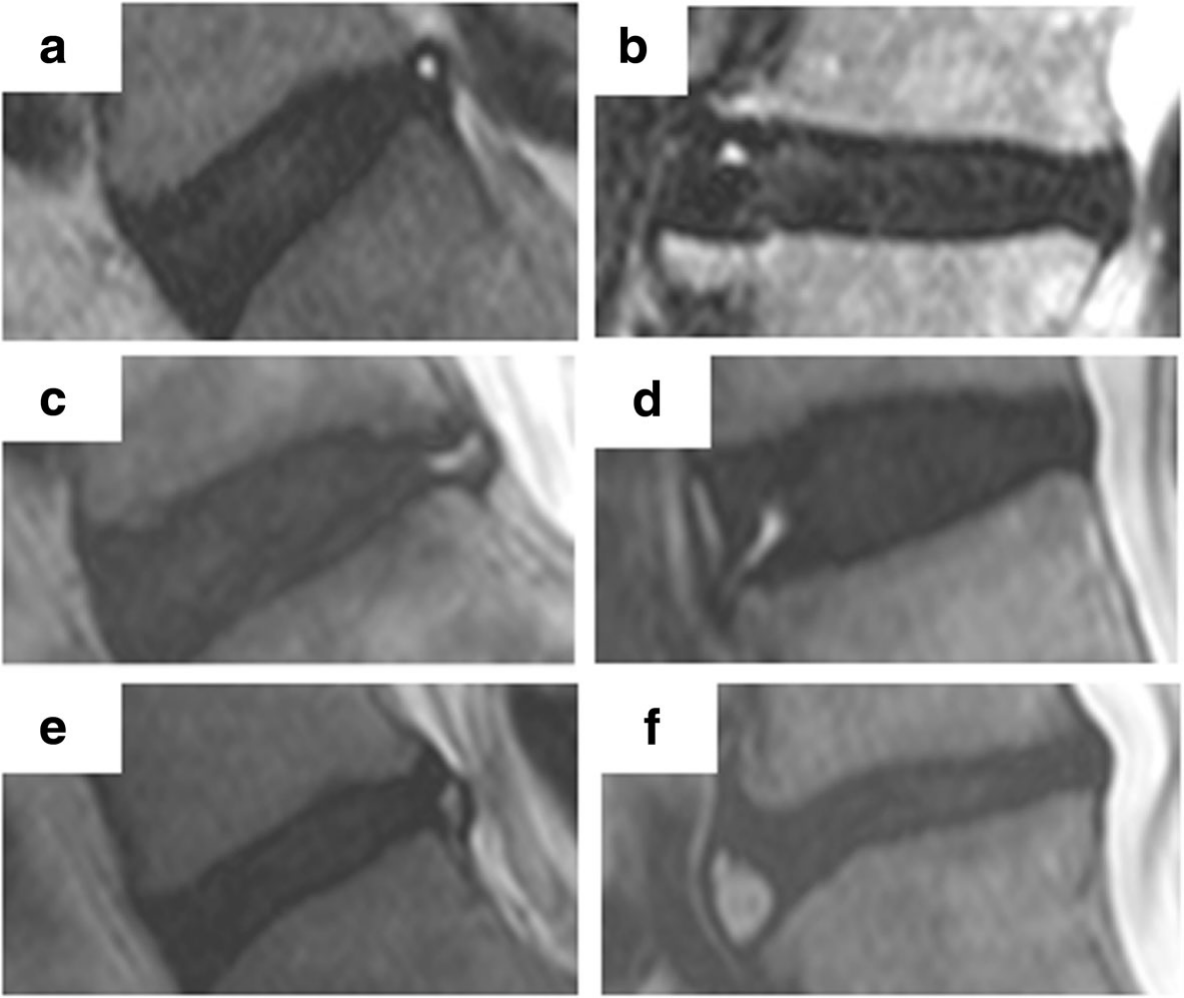
Novel classification of HIZ on T2-weighted MRI by Teraguchi et al. **a** Posterior round type. **b** Anterior round type. **c** Posterior fissure type. **d** Anterior rim type. **e** Posterior vertical type. **f** Anterior enlarge type

To better understand the clinical implications and pathogenesis of HIZs, the standard phenotype definition must be better characterized and classified. The prevalence of HIZs has been widely variable in previous studies due to difficulty of recognizing HIZs [19–31]. With modern MRI technology, novel sequences allow better image acquisition and higher resolution so that HIZ can be easier identified as annular tears in any part of the annulus. Teraguchi et al. [12] suggested HIZ new phenotypes were classified based on six shape types based on the anterior and posterior intervertebral disc (anterior and posterior round type, posterior fissure type, posterior vertical type, anterior rim type, and anterior enlarged type) (Fig. 4). Therefore, a standard classification is more precise and comprehensive, and can be utilized for any future analysis regarding phenotype association and clinical relevance research.

Systematic literature reviews offer an excellent opportunity to gain an overview of challenging topics. However, they also have some limitations that need to be addressed. Most abstracts and studies were screened in this review, but a small number of relevant studies make interpretation of any data a challenge. Due to our strict inclusion criteria, there were only six studies included for review. Finally, there is always the danger of publication bias, particularly if the subject is new and perhaps controversial. Nonetheless, what our review stresses is that there is a paucity of high level of evidence studies addressing the clinical relevance of HIZs of the lumbar spine. Larger studies with in-depth statistical analyses of the HIZs in relation to disc levels and patterns therein are further needed to assess the true impact on pain development.

## Conclusion

Since the original description of the HIZs in the 1990s, considerable interest has surrounded this spinal phenotype. Our systematic review has noted that HIZs of the lumbar spine may be related to pain; however, uniform consensus is not noted among studies. A more detailed investigation of the underlying pathology and topography/morphology of HIZs could be essential as well as its clustering with various spinal phenotypes can provide a unique complex phenotypic variant of this imaging finding. If found to be clinically relevant, this potential biomarker can be standardized for future clinical and research initiatives. These include correlating HIZs with symptoms, validating with cross-ethnic and cross-cohort studies, and potentially expanding its role as a pain biomarker in future initiatives. Therefore, utilizing a multimodal MRI approach to better characterize the HIZ phenotype is imperative to assist communication between study centers and aid large-scale analyses. Such information can be instrumental in predictive modeling as well as future omics studies.

## Abbreviations

HIZs: High-intensity zones
LBP: Low back pain
MRI: Magnetic resonance imaging
